# Purple urine bag syndrome in neurological deficit patient: A case report

**DOI:** 10.1016/j.ijscr.2023.107953

**Published:** 2023-02-24

**Authors:** Pande Made Wisnu Tirtayasa, Ronald Sugianto, Isabella Valentina, Alwyn Geraldine Samuel

**Affiliations:** a Department of Urology, Faculty of Medicine, Universitas Udayana, Universitas Udayana Teaching Hospital, Bali, Indonesia; b Medical Doctor Study Program, Faculty of Medicine, Universitas Udayana, Bali, Indonesia; c Department of Clinical Pathology, Tarakan Regional Hospital, Jakarta, Indonesia; d Department of Surgery, Tarakan Regional Hospital, Jakarta, Indonesia

**Keywords:** Purple urine bag syndrome, Neurogenic bladder, Paraparesis, Congenital rubella infection

## Abstract

**Introduction and importance:**

Purple urine bag syndrome (PUBS), described first in 1978, is a rare phenomenon with purplish discolorations in the urine collecting bag. This report aims to provide a general overview of PUBS, its pathogenesis, and the recommended treatments.

**Case presentation:**

A woman patient, 27 years old, with prior history of congenital rubella infection complained of urinary retention. The patient routinely had foley catheterization due to neurogenic bladder accompanied by paraparesis inferior for 1.5 years. She also suffered bilateral lower extremities edema with infected wounds for two weeks, which showed a purple urine color in the urine bag. The laboratory examination demonstrated iron deficiency anemia, hypokalemia, and blood alkalosis.

**Clinical discussion:**

The cause of purplish discolorations of PUBS is the mixing of indigo, blue pigment, and indirubin, red pigment, which are results of dietary digestion, hepatic enzymes, and bacterial urine oxidation. The main risk factors are female patients, constipation, older age, recurrent UTI, renal failure, and urinary catheterization, dominantly on chronic treatment with polyvinyl chloride (PVC) urinary catheter or bag.

**Conclusion:**

The management should be promptly, rigorously, and appropriately because the complicated UTI has a high-risk progression of urosepsis.

## Highlights

• Purple urine bag syndrome is a rare medical phenomenon.
• Purplish discolorations are shown by mixing indigo and indirubin.
• The main risk factors are female, constipation, and urinary catheterization.
• The main etiology of urosepsis was gram-negative bacteria, similar to PUBS.
• PUBS management has to be appropriate due to high morbidity and mortality.

## 1 Introduction and importance

Purple urine bag syndrome (PUBS) is a phenomenon of purplish discolorations in the urine collecting bag, which remains underdiagnosed. PUBS described first in 1978 by Barlow and Dickson [1], has been associated with constipation, urinary tract infections (UTI), renal failure, and urinary catheterization, dominantly on chronic treatment [2], [3]. Even though the urine is relatively clear, the cause of purplish discolorations of the tube or collecting bag is the mixing of indigo, blue pigment, and indirubin, red pigment, which are results of dietary digestion, hepatic enzymes, and bacterial urine oxidation [3], [4]. This report aims to provide a general overview of PUBS, its pathogenesis, and the recommended treatments.

## 2 Case presentation

A woman patient, 27 years old, with prior history of congenital rubella infection complained of urinary retention. The patient routinely had foley catheterization due to neurogenic bladder accompanied by paraparesis inferior for 1.5 years. She also suffered bilateral lower extremities edema with infected wounds for two weeks. The patient's vital signs and physical examinations were hypotension, tachypnea, and hypoxia, and the bladder was full. The following laboratory examination demonstrated hemoglobin 9.2 g/dL with microcytic and hypochromic erythrocytes, serum iron 14 μg/dL, ferritin, 4.45 ng/mL, potassium 2.9 mEq/L, blood urea nitrogen 14 mg/dL, serum creatinine 0.4 mg/dL, blood pH 7.65, PaCO2 20.2 mmHg, PaO2 212.3 mmHg, leucocytes urine sedimentation 5–8 LPF. Therefore, the patient was diagnosed with iron deficiency anemia, hypokalemia, and blood alkalosis.

**Fig. 1:**
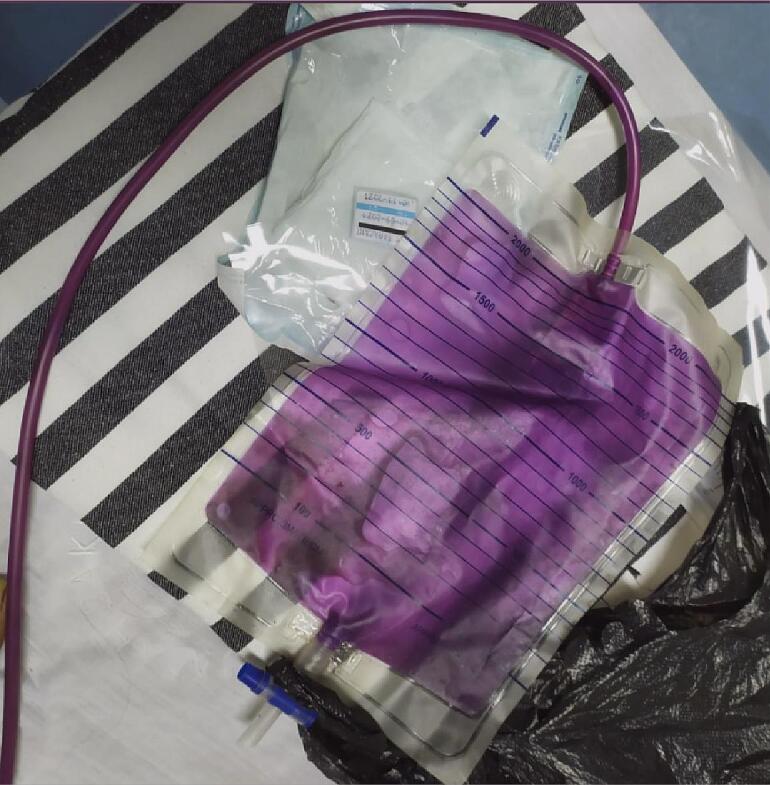
Purple urine in collecting bag. (For interpretation of the references to color in this figure legend, the reader is referred to the web version of this article.)

Based on those data, the patient underwent emergency treatment with 15 L of oxygen per minute by non-rebreathing mask, rehydration, empirical antibiotics, and foley catheterization replacement. After that, a purple urine color was found in the urine bag (Fig. 1). Then, urine culture yielded *Escherichia coli*, *Pseudomonas aeruginosa*, and *Klebsiella pneumonia*, gram-negative bacteria sensitive to meropenem, so the antibiotic was changed. The disappearance of purple discoloration in the urine bag and the patient becoming asymptomatic after one week of treatment. This case presentation followed SCARE Guideline Checklist 2020 [5].

## 3 Clinical discussion

The pathogenesis of PUBS (Fig. 1) initiates by the tryptophan in the bowel from the gastrointestinal process, digestion, and absorption. The bacteria in the intestine generated indoxyl sulfate from tryptophan metabolism [6]. Then, hepatic enzymes turned into the conjugate indoxyl sulfate form and secreted into the kidney. In the urinary tract, the conjugate indoxyl sulfate was metabolized to indoxyl through the complete oxidation of gram-negative bacteria phosphatases and sulfat**as**es, which convert into indigo and indirubin. Then, purplish discolorations were shown by mixing indigo, blue pigment, and indirubin, a red pigment [3], [4], [7]. The most common bacteria related to PUBS were: *E. coli* 20.8 %, *Proteus mirabilis* 16.2 %, and *Klebsiella pneumonia* 13,6 % [8].

The purple discoloration of urine is known to be associated with multiple risk factors. The main risk factors are female patients, constipation, older age, recurrent UTI, renal failure, and urinary catheterization, dominantly on chronic treatment with polyvinyl chloride (PVC) urinary catheter or bag [7], [8] followed by dementia, prolonged bed rest, institutionalization, dehydration, high urinary bacterial counts and alkaline urine [1], [8]. In this report, the patient had four main risk factors followed by three additional risks.

The main etiology of urosepsis is gram-negative bacteria, similar to bacteria related to PUBS, as follows: *E. coli* 50 %, *Proteus* spp. 15 %, *Enterobacter* and *Klebsiella* 15 %, *P. aeruginosa* 5 %, and the others are gram-positive bacteria [9]. For a quick and accurate diagnosis, the quick Sequential Organ Failure Assessment (qSOFA) scoring system is mandatory, including altered mental status (GCS < 15), compromising respiratory rate (>22/min), and systolic blood pressure of 100 mmHg or less [10]. Our patient's urine cultures yielded the same bacteria with two qSOFA scores.

Since there is no guideline for PUBS management, the treatment of PUBS has varied according to the pathogenesis and the patient's condition. Moreover, the treatment should be appropriate due to high morbidity and mortality [11]. Based on our case, we recommend stabilizing the vital sign and treating the systemic disease first, such as oxygenation and intravenous rehydration. Afterward, the empirical antibiotics and the catheter replacement should be done, and non-plastic catheter bags could be an alternative [7]. The broad spectrum of antibiotics therapy that covers extended-spectrum was recommended [6]. The antibiotic switching can be performed once the urine culture and antibiotic sensitivity results showed up.

## 4 Conclusion

The complicated UTI has a high-risk progression of urosepsis. The management should promptly, rigorously, and appropriately, including the specified antibiotics for UTI infections, replacing the catheter, and the prevention of recurrence or persistence of the PUBS phenomenon.

## Consent

The informed consent was written by the patient in the Indonesian language for further publication of this case report anonymously. A copy of the written consent is available for review by the Editor-in-Chief of this journal on request.

## Source of funding

None.

## Ethics committee approval

Not applicable.

## Author contribution

Pande Made Wisnu Tirtayasa: Conceptualization; Formal analysis; Funding acquisition; Investigation; Methodology; Project administration; Resources; Supervision; Validation; Visualization.

Ronald Sugianto: Roles/Writing - original draft; Writing - review & editing, Conceptualization; Formal analysis; Investigation; Methodology; Project administration; Visualization.

Isabella Valentina: Data curation; Funding acquisition; Investigation; Resources; Software; Supervision; Validation; Visualization.

Alwyn Geraldine Samuel: Performed the procedure, Data curation; Funding acquisition; Investigation; Resources; Supervision; Validation; Visualization.

## Research registration

None.

## Declaration of competing interest

None.
